# Tackling protozoan parasites of cattle in sub-Saharan Africa

**DOI:** 10.1371/journal.ppat.1009955

**Published:** 2021-10-14

**Authors:** Paula MacGregor, Vishvanath Nene, R. Ellen R. Nisbet

**Affiliations:** 1 School of Biological Sciences, University of Bristol, Bristol, United Kingdom; 2 International Livestock Research Institute, Nairobi, Kenya; 3 School of Bioscience, University of Nottingham, Nottingham, United Kingdom; University of Wisconsin Medical School, UNITED STATES

## Introduction

**Fig 1:**
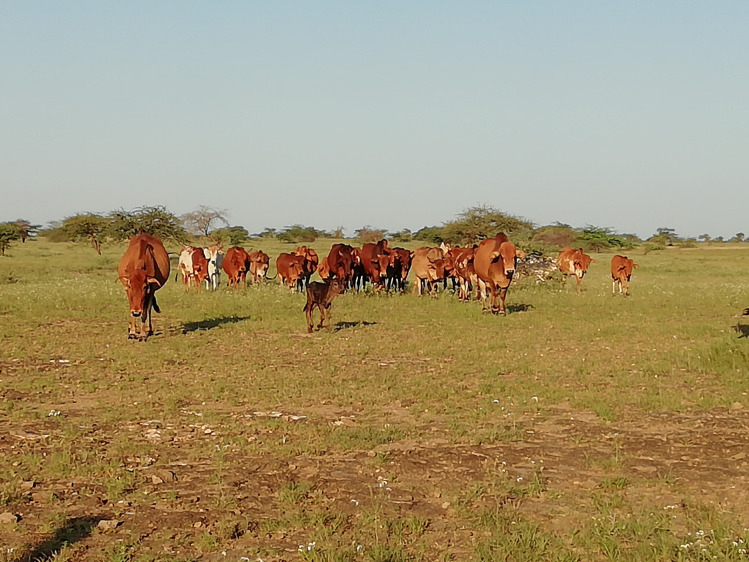
Cattle in rural Kenya. *Photograph from February 2020*, *taken by R*. *E*. *R*. *Nisbet*.

Cattle are an incredibly valuable asset to farmers throughout the world (Fig 1). They provide power, transport, fertiliser, fuel, and nutrition. In some areas, cattle guarantee a family’s food and economic security and act as important indicators of social status. In Africa, there are estimated to be over 360 million cattle, an increase of 22% between 2010 and 2019 [1]. As the continent continues its population and economic growth, improved productivity and sustainable growth of livestock will be required to meet the demand for food while mitigating their negative impacts.

Cattle are host to a plethora of infectious agents including viruses, bacteria, prions, and a range of parasites comprising worms, ectoparasites, and protozoa. Many cattle pathogens are closely related to pathogens of humans, including some that are zoonotic or with zoonotic potential. Any infectious disease that causes loss of cattle life or decreased productivity (work, growth, or fertility) imposes an economic impact. This burden heavily and disproportionately affects low- and-middle-income countries and, in particular, smallholder farmers and pastoralists. Among the myriad of infectious agents of cattle in sub-Saharan Africa, there are a small selection of protozoan pathogens that collectively cost the region’s economy billions of US$ per annum. These are some of the biggest constraints to livestock production across sub-Saharan Africa, affecting food security and hindering socioeconomic development.

Here, we examine the current situation and ongoing progress made in tackling these diseases. Typically, human-infective parasites are subject to more experimental research than relatives that infect cattle. We highlight the discrepancy in our knowledge and research capacity between human and veterinary parasites. This research gap needs to be addressed if the effects of such pathogens on livestock are to be more effectively prevented. We describe the improved tools and resources needed so that these parasitic diseases can be studied effectively.

### Which protozoan diseases impact cattle farming in sub-Saharan Africa?

The parasites that cause the most significant protozoan diseases in cattle in sub-Saharan Africa belong to order Kinetoplastida (phylum Euglenozoa) and the phylum Apicomplexa, taxa which encompass some of the world’s most devastating disease-causing parasitic species for humans, livestock, and crops.

#### Kinetoplastid pathogens of cattle in sub-Saharan Africa

The African trypanosomes *Trypanosoma congolense*, *Trypanosoma vivax*, and, to a lesser extent, *Trypanosoma brucei* are the causative agents of bovine animal African trypanosomiasis (AAT) or nagana. These parasites are also able to infect a number of livestock, e.g., small ruminants and camels, and are transmitted via a tsetse fly vector (*Glossina* spp.). Symptoms of acute bovine AAT include fever, anaemia, and weight loss and can be fatal. Most cases, however, develop into chronic disease associated with weakness, neurological symptoms, and reduction in milk production and fertility. *T*. *congolense* and *T*. *brucei* are geographically restricted to the “tsetse belt” across sub-Saharan Africa, although *T*. *vivax* has additionally established in South America through mechanical transmission by stable flies (*Stomoxys*) and horse flies (*Tabanids*). Given the worldwide distribution of these latter fly species, it is possible that that the geographical range of *T*. *vivax* could expand. While *T*. *brucei* is the least pathogenic of the 3 bovine AAT-causing species of African trypanosomes, 2 of the 3 subspecies of *T*. *brucei* are human infective, causing human African trypanosomiasis (HAT). More distantly related human-infective kinetoplastids include *Trypanosoma cruzi*, which causes Chagas disease in South America and over 20 *Leishmania* species that cause leishmaniasis.

#### Apicomplexan pathogens of cattle in sub-Saharan Africa

The Apicomplexa are a large group of intracellular pathogens. In humans, diseases resulting from apicomplexan infection include malaria, toxoplasmosis, and babesiosis. Across sub-Saharan Africa, the most important veterinary apicomplexans for cattle are 2 piroplasma species: *Theileria parva*, the causative agent of the East Coast fever (ECF), and *Babesia bovis* and *Babesia bigemina*, causative agents of bovine babesiosis (Table 1). Other apicomplexan species cause tropical theilerioses, cryptosporidiosis, toxoplasmosis, neosporosis, besnoitiosis, and eimeriosis in cattle.

**Table 1:** Research methods used for the study of human disease–causing parasite species that could be applied to cattle disease–causing parasites [2–9]. Note that the nonhuman infective subspecies of *T*. *brucei* (*T*. *b*. *brucei*) is the most widely used African trypanosome in the laboratory, acting as a model system for the human-infective subspecies *T*. *b*. *rhodesiense* and *T*. *b*. *gambiense*. Example studies are not exhaustive of the available literature.

		Kinetoplastids	Apicomplexans	
**Disease**	**Diseases caused** Disease causing species / sub-species in cattle	AAT *Trypanosoma congolense*, *Trypanosoma vivax*, *Trypanosoma brucei brucei* (minor role in cattle disease)	East Coast Fever *Theileria parva*	Bovine Babesiosis *Babesia bovis* *Babesia bigemina*
	**Closely related** human disease (and pathogen species / sub-species)	HAT (*T*. *brucei gambiense* and *T*. *brucei rhodesiense*) Chagas disease (*T*. *cruzi*) Leishmaniasis (*Leishmania species*)	Malaria (*Plasmodium* species) Toxoplasmosis (*Toxoplasma gondii*) Human Babesiosis (*Babesia* species)	
**Research** capabilities	Key database	TriTrypDB.org	PiroplasmaDB.org	
	**Whole-cell studies** Genomics, bioinformatic, transcriptomics and proteomics	As these methods can be carried out without GM, it is somewhat straightforward to conduct genomics, bioinformatics, transcriptomics and proteomics (whole cell or sub-cellular quantitative mass spectrometery) on any species where sufficient interest, resources and starting material allows. Note that due to differential gene expression between parasite life-stages, often studies are comparative.		
	*Examples*	Genomes are available for *T*. *congolense* and *T*. *vivax*, although are less heavily curated than for *T*. *brucei*. There are five whole-cell proteomics datasets for BSF *T*. *brucei* available at TriTrypDB.org, providing evidence of expression of over 5000 proteins in the BSF. Only one such study is available for *T*. *congolense*, providing evidence of expression of ~2000 proteins in BSFs. To date, there are no sub-cellular proteomics studies on *T*. *congolense*. Methods developed for *T*. *brucei* should be transferable, albiet with optimisation.	Genomes and whole cell transcriptomes are available for *T*. *parva*, *B*. *bovis* and B. bigemina. Only *T*. *parva* has been subject to whole-cell quantitative proteomics, in the sporozoite life-stage. Alongside standard whole-cell transcriptomics, there have been a range of single-cell transcriptomic studies carried out in *Plasmodium* sp. and *Toxoplasma gondii*. There are a number of whole-cell and sub-cellular proteomics studies for *Plasmodium* sp. and *T*. *gondii*. Recently, a whole-cell spatial proteome was determined for *T*. *gondii* providing localisation assignments for over 1900 proteins.	
	**Experimental tractability** Laboratory culture, GM and genome-wide studies	In vitro cell culture and GM is routine for *T*. *brucei* BSFs, including RNAi. In vitro culture of *T*. *congolense* BSFs is possible for one isolate and there has been significant developments in tools for growth and GM in recent years, vastly improving experimental tractability of this species. Equivalent methods for *T*. *vivax* still lag behind.	In vitro cell culture established for *T*. *parva* schizont stage. No genetic modification is yet possible; establishing a stable GM methodology is a priority.	In vitro culture of *B*. *bovis* is established. Stable GM is possible, including CRISPR/Cas9 genome editing. No genome-wide studies have been published.
	*Examples*	Genome wide RNAi and gain-of-function studies have been conducted in *T*. *brucei*, providing phenotypic data on over 7000 genes in each case.	A genome wide CRISPR screen has been carried conducted for *T*. *gondii* providing phenotypic data (fitness score) for over 8000 genes.	

AAT, animal African trypanosomiasis; BSF, bloodstream form; ECF, East Coast fever; GM, genetic modification; HAT, human African trypanosomiasis; RNAi, RNA interference.

*T*. *parva* is transmitted by ticks (*Rhipicephalus appendiculatus*), which feed on the blood of cattle and other mammals. During tick feeding, *T*. *parva* sporozoites enter into the cattle bloodstream. The extracellular sporozoites attach and enter host cells, primarily lymphocytes. Once inside, the parasite rapidly dissolves the host-derived parasitophorous vacuole membrane (PVM) and proliferates as an intracellular schizont in the host cell cytoplasm. The parasite immortalises the infected host cells, resulting in a cancer-like uncontrolled proliferation [10]; the molecular basis for this remains mostly uncharacterised. The cattle suffer diarrhoea, fever, anorexia, and laboured breathing. In 1999, it was estimated that stock losses to ECF were 1 million cattle [11]. It is reasonable to assume that annual losses have increased with cattle numbers. *T*. *parva* originated in the wild African buffalo (*Syncerus caffer*) population, where the parasite is ubiquitous but does not typically cause disease.

Bovine babesiosis is most commonly found in tropical and subtropical countries, especially in sub-Saharan Africa and South America. Two main species cause disease in African cattle, *B*. *bovis* and *B*. *bigemina*, and are transmitted by *Rhipicephalus* ticks. Following tick bite, the parasites invade host red blood cells, causing anaemia and fever, and infection can also lead to cerebral babesiosis (*B*. *bovis* only) and death of cattle. A recent South African survey revealed that up to three-quarters of cattle may be infected, dependent on region [12]. Although these cattle may appear healthy, they can have decreased milk and meat production, as well as acting as carriers for transmission [12].

### What preventions and treatments are available against these protozoan infections in cattle?

Efforts to control parasitic disease are hindered by the challenge of implementing vector control strategies across the vast expanses of tsetse and tick-populated land; emerging drug resistance and the prevalence of counterfeit drugs; and a lack of suitable vaccination programmes.

Tick and tsetse fly control are used to prevent against infections, as has been the case for many generations. Vector control is complex and comes with many limitations, including the following: (i) Tsetse- and tick-infected regions are vast; therefore, traps can only provide a local level of protection, which needs to be ongoing; (ii) ticks and related insects are a valuable source of nutrition to reptiles and birds and so large-scale insecticide use is not feasible; (iii) cattle plunge-dipping into toxic organophosphates or synthetic pyrethroids can cause significant illness to the farmer and the environment and is not a widely available control option; and (iv) the choice of insecticides used are key, due to selective toxicity and resistance [13].

Vaccination of cattle to prevent disease transmission is therefore considerably preferable to arthropod management. Although ECF and babesiosis are both preventable diseases through live cell vaccination, such vaccines require a cold chain from lab to cow, which is inappropriate for use in rural settings due to expense and logistics. Additionally, vaccination with *T*. *parva* is followed by antibiotic treatment in a simultaneous infection treatment immunisation model. These impracticalities mean that the vaccine is not widely used. A modern subunit, RNA or DNA vaccine, is urgently required [14].

No vaccine against AAT exists and has long been thought unlikely to be developed. This is largely due to the parasites immune evasion strategy of antigenic variation as well as the apparent lack of natural capacity for cattle to clear infection, despite the presence of antibodies to nonvariant surface proteins. However, an AAT vaccine that could prevent establishment of the infection would be hugely valuable, and recent work has identified a well-conserved *T*. *vivax* vaccine target, against which vaccination in a murine model results in long-term sterile immunity [15].

The primary drug available to treat ECF is buparvaquone; this is over 30 years old and expensive to use, yet, to our knowledge, no new drugs are under development. Buparvaquone is also used to control *Theileria annulata* (causative agent of tropical theileriosis), and resistance due to mutations in the cytochrome b gene has been identified. The primary treatment for babesiosis is imidocarb. Concerns have been raised regarding use of imidocarb in livestock due to its passage into milk and retention in tissues that are then used as human food [16]. Currently, AAT is primarily controlled by regular administration of prophylactic isometamidium chloride and therapeutic diminazene aceturate and homidium bromide/chloride. The latter is a mutagenic (possibly carcinogenic) DNA intercalating agent, which can be toxic at high doses. Benzoxaboroles have been identified as a potential new class of veterinary drugs against AAT (for example, see [17]), with research and development underway.

### Can the developments in research on human-infective protozoa inform our understanding of cattle parasites?

Typically, human-infective parasites are subject to more experimental research than relatives that infect cattle. This research gap needs to be addressed if the effects of such pathogens on livestock are to be more effectively prevented. The cellular and infection biology of cattle-infecting parasites do vary from human-infective species and so cannot always be reliably inferred. For example, many apicomplexan parasites are motile and invade host cells using an apical complex, forming a pointed end to the cell [18]. In contrast, *Theileria* parasites are nonmotile and do not reorient during host cell invasion. These differences are fundamental when designing subunit vaccines against cell surface proteins. Similarly, while the mutations that lead to diminazene aceturate resistance have been characterised in *T*. *brucei*, the mode of resistance in *T*. *congolense* and *T*. *vivax* is both distinct and unknown [19,20]. However, many of the tools and techniques developed for human-infective pathogens can be adapted for use in animal pathogens.

Table 1 describes some of the important issues that require addressing. For African trypanosomes, this includes the need to establish culturing techniques for *T*. *vivax*, improved culture systems for *T*. *congolense* (especially to permit growth of additional bloodstream form strains) and established robust genetic modification protocols, progress for which has been recently made [3]. For the apicomplexans, in vitro culture and stable genetic modification of *B*. *bovis* are possible, but only one life stage of *T*. *parva* can be grown in vitro, with no ability to genetically modify to date. For all of the species discussed here, whole-cell proteomics and genome-wide knock-out studies, akin to those previously carried out in related human-infective species, would be extremely beneficial. A combination of improved experimental tractability and large datasets would provide a step change in the capacity to study these veterinary important organisms.

While conscious of biological differences, advances in human-infective parasitology research can and should be exploited wherever possible to improve research capacity and knowledge of cattle parasites. For example, related parasite species often have similar capacity for genetic modification, so experimental protocols (i.e., transfection methodology) and resources (i.e., plasmids) developed in one species can be the starting point for developing methods for others. Similarly, the advancement of experimental techniques in one species (i.e., optimisation of cell fractionation methods for subcellular proteomics) may then facilitate the use of that technique in related organisms. Where novel biology is uncovered, the assessment of similar features (i.e., conserved protein function) in related species is less resource demanding than the original discovery. Finally, the development of treatments or prevention measures against human-infective species could have real impact on cattle diseases if they were studied or developed in parallel.

There is no doubt that veterinary important species continue to be understudied compared to their human-infective counterparts and that we cannot simply extrapolate data from one species to another. However, this is progressively being recognised as an important area of research and with the ability to draw on data and methodology from human-infective parasite species, the scientific community is well placed to start to close this gap.
